# Expression analysis of blood samples shows elevated 5′-tRF-His-GTG in breast cancer patients

**DOI:** 10.1038/s41598-025-19458-w

**Published:** 2026-05-15

**Authors:** Mohammad Salehi, Mohaddese Mohsenipour, Fatemeh Ghadimi, Marie Saghaeian Jazi, Shakur Babaei, Mohammad Javad Kamali, Mohammad Shafiee, Ali Akbar Saffar Moghadam

**Affiliations:** 1 Department of Medical Genetics, School of Advanced Technologies in Medicine, Golestan University of Medical Sciences, Gorgan, Iran; 2 Metabolic Disorders Research Center, Golestan University of Medical Sciences, Gorgan, Iran; 3 Department of Medical Genetics, School of Medicine, Babol University of Medical Sciences, Babol, Iran; 4 Cancer Research Center, Golestan University of Medical Sciences, Gorgan, Iran

**Keywords:** Non-coding RNA, Breast cancer, tRFs, Bioinformatics, Breast cancer, Gene expression, Medical genetics, Cancer, Cell biology, Genetics, Molecular biology, Medical research, Molecular medicine, Oncology

## Abstract

tRNA-derived fragments (tRFs) have emerged as promising non-invasive biomarkers for breast cancer. Understanding their diagnostic potential is essential for improving early detection and patient outcomes. This study aimed to investigate the expression level of the 5′-tRF-His-GTG in the blood of breast cancer patients and assess its potential as a diagnostic or prognostic biomarker. Candidate tRFs were identified through bioinformatic analyses using MINTbase, MODOMICS, and BBcancer, focusing on expression likelihood in biofluids and chemical modification profiles. A total of 56 blood samples, including 28 from breast cancer patients and 28 from healthy controls, were collected. Total RNA was extracted, and cDNA was synthesized via reverse transcription. Quantitative real-time PCR was performed to assess the expression level of tRF-32-XSXMSL73VL4YK. Statistical analysis was conducted using an independent t-test with a significance threshold of *p* < 0.05. tRF-32-XSXMSL73VL4YK, characterized by minimal chemical modifications, was selected for further investigation. It was significantly upregulated in breast cancer patients compared to healthy controls (*p* < 0.001). However, no significant associations were found between its expression and clinicopathological features such as age, tumor size, TNM stage, or IHC markers (*p* > 0.05). Our study identifies tRF-32-XSXMSL73VL4YK as a significantly upregulated 5’tRF-His-GTG in breast cancer, yet its expression shows no correlation with key clinical characteristics. While tRF-32-XSXMSL73VL4YK may not serve as a standalone diagnostic marker due to its lack of correlation with clinical features, its consistent upregulation suggests promise as a component of a multi-marker panel or as a prognostic biomarker.

## Introduction

Breast cancer stands as the most prevalent form of cancer among women, constituting approximately 25.2% of all female cancer cases reported each year. In 2022, approximately 2.3 million new cases were identified, and breast cancer was responsible for 670,000 deaths worldwide, ranking it as the fifth leading cause of cancer-related mortality^1,2^. Looking ahead, the global burden of breast cancer is expected to escalate significantly, with incidence projected to increase by 38% and mortality by 68% by 2050 if current trends continue^3,4^. The development of breast cancer in women can be influenced by various factors, including genetic predisposition, family history, age, and environmental factors^5^. In a meta-analysis study, the prevalence of breast cancer among Iranian women was determined to be 23.6% based on a sample size of 39,596 patients^6^.

Despite recent advancements in early diagnosis and effective treatments, some patients still progress to metastatic disease after therapy, often without a clear understanding of the underlying reasons^7^. The 5-year survival rate for metastatic breast cancer is only 28%, markedly lower than the 86–99% survival rates observed in women with localized or regional breast cancer^8^. This stark contrast underscores the critical importance of identifying patients at early and treatable stages of the disease to reduce breast cancer mortality.

Currently, breast cancer diagnosis relies on multiple methods, including clinical examinations, mammography, serological biomarkers, ultrasound, immunohistochemical (IHC) markers (such as ER, PR, HER2, Ki67), MRI, and other imaging modalities^9,10^. Although these approaches have significantly improved early detection and treatment planning, they also present limitations in accuracy, early diagnosis, and invasiveness. For instance, mammography is hampered by high false-positive and false-negative rates, reduced sensitivity in detecting small tumors, challenges in women with dense breast tissue, and concerns related to radiation exposure^11^. In contrast, biofluid-based tests offer several advantages: they are non-invasive, convenient, highly sensitive, and cost-effective. In contrast, biofluid-based tests offer several advantages over imaging and biopsy methods for cancer detection. These tests are convenient, non-invasive, highly sensitive, and cost-effective^12^. Hence, developing novel, specific, and sensitive screening panels through the identification of non-invasive biomarkers is critical for early intervention when treatment options are most effective^2^.

The limited specificity and sensitivity of conventional serological biomarkers—such as carbohydrate antigen 153 (CA153), cancer antigen 125 (CA125), CA27.29, and carcinoembryonic antigen (CEA)—have raised concerns about their overall effectiveness^13^. To improve treatment outcomes and avoid unnecessary procedures, it is imperative to identify novel biomarkers with greater accuracy and reliability. Non-coding RNAs (ncRNAs) have shown promise as molecular markers for the diagnosis, prognosis, and treatment of various diseases, including breast cancer^14^. Different classes of ncRNAs—including microRNAs, long non-coding RNAs, circular RNAs, Piwi-interacting RNAs, and tRNA-derived fragments (tRFs)—regulate gene expression at the epigenetic, transcriptional, and post-transcriptional levels through mechanisms such as methylation, histone modification, chromatin remodeling, or competitive inhibition^15^.

Over the past decade, research has revealed a unique category of small ncRNAs known as tRFs. Contrary to earlier assumptions that tRFs were random degradation products of tRNAs, they are now understood to be produced through the specific cleavage of pre-tRNAs or mature tRNAs by dedicated endonucleases^16,17^. tRFs are classified into distinct groups based on their length and origin, including tRF-1s, 3′-tRFs, 5′-tRFs, internal tRFs (i-tRFs), and tRNA halves (tiRNAs), such as 5′-tiRNA and 3′-tiRNA^18,19^. Widely distributed across multiple cell types and tissues, tRFs play significant roles in physiological processes by functioning similarly to miRNAs, guiding RNA-binding proteins, participating in epigenetic regulation, contributing to ribosomal protein synthesis, and interfering with translation initiation^20,21^. Compelling evidence indicates that the dysregulation of tRFs is a key mechanism through which tumor cells influence various oncogenic processes^22^.

tRFs have emerged as promising diagnostic and prognostic biomarkers, particularly in the early stages of diseases due to their unique characteristics^23,24^. Notably, tRFs exhibit tissue-specific expression patterns and can be detected in various biofluids, including serum, plasma, saliva, and urine. Noteworthy clinical correlations have been observed between changes in tRF levels in these biofluids and important patient outcomes^25,26^. Recent discoveries of tRFs in exosomes further support their potential utility in liquid biopsies, enabling the analysis of molecular signatures from cancer cells^27,28^. Moreover, the extensive chemical modifications of tRFs render them resistant to degradation, ensuring their stability in clinical samples^25,28^. This stability, combined with their capacity to complement traditional biomarkers (e.g., protein markers and genetic mutations), enhances the sensitivity and specificity of breast cancer diagnosis by offering a more comprehensive diagnostic approach^29^. Consequently, tRFs hold great promise as non-invasive biomarkers for both diagnostic and prognostic applications.

Recent studies have observed differential expression levels of circulating tRFs between breast cancer patients and healthy individuals, further supporting their potential as diagnostic and prognostic indicators^30,31^. Previous research has reported that various fragments derived from tRNA-His can play dual roles as tumor suppressors or oncogenes, highlighting their complex involvement in tumorigenesis^32–34^. Therefore, this study aimed to investigate the expression levels of a 5′ tRF-His-GTG in the blood of breast cancer patients compared to matched healthy controls, assessing its potential as a biomarker capable of distinguishing cancer patients from healthy individuals.

## Materials and methods

### Screening and identification of the cancer associated-tRFs using bioinformatic analysis

A bioinformatic approach was employed to screen and identify potential tRFs and their involvement in cancer, with a particular focus on tRNA-His and its fragments. The first step was to ensure accurate amplification of tRFs using quantitative reverse transcription PCR (qRT-PCR). To achieve this, it was crucial to identify all potential modifications present on the tRF fragment. The MODOMICS database was utilized for this purpose, which provided information on modifications associated with tRNA-His and its corresponding fragments. To investigate the expression profile of the selected tRF, various databases such as MINTBase V2.0 (with 12,023 analyzed datasets) and BBCancer (with overall 7184 samples of cancer tissues, including 788 breast cancer tissues) were examined^35,36^. These databases contained information on the tRF expression profiles in different types of cancers. This study investigated expression levels of tRF-32-XSXMSL73VL4YK in blood samples from patients to lay ground for further investigations on whether this tRF can be considered for potential future biomarker.

### Samples and patients

**Fig. 1:**
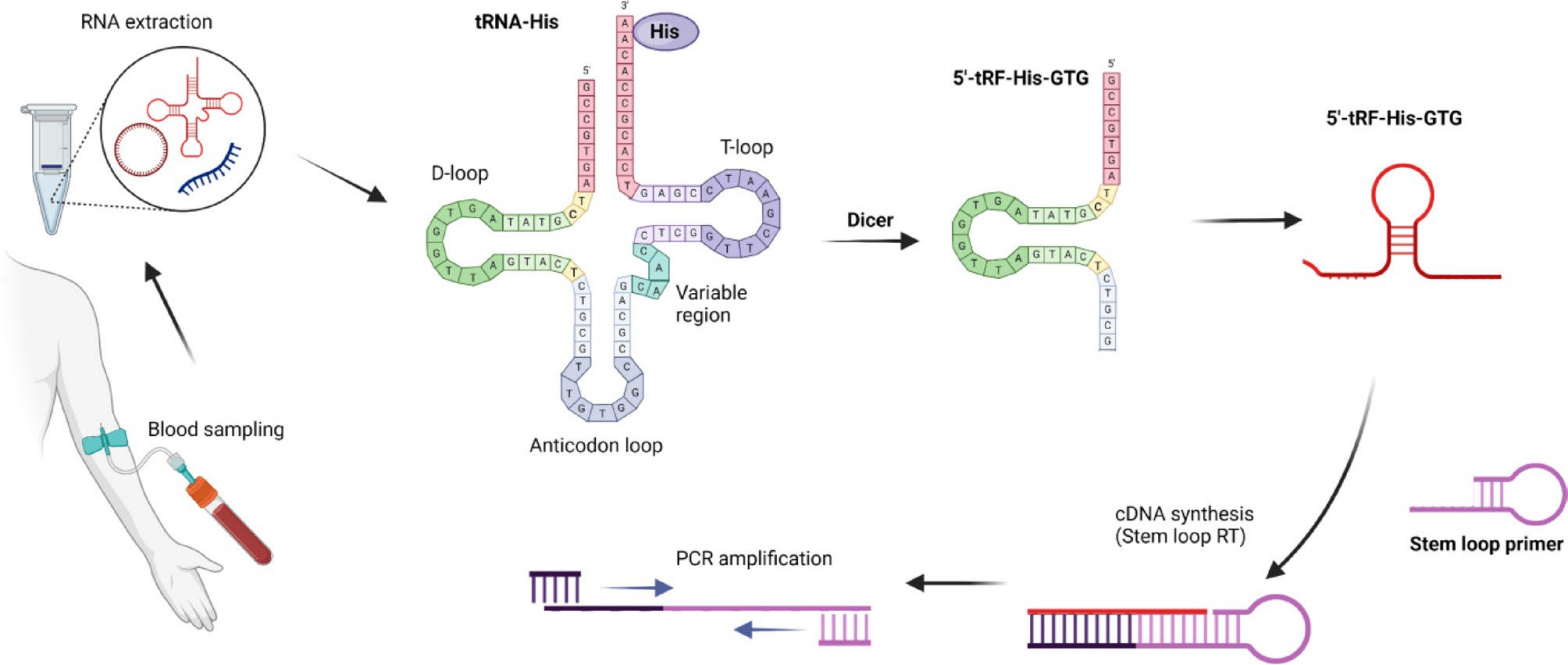
A schematic illustration of the study’s workflow.

The blood samples used in this study were obtained from the 5th Azar Hospital in Gorgan, Iran. The research included a total of 56 blood samples, consisting of 28 patients diagnosed with breast cancer between 2022 and 2023, and 28 healthy controls. We matched each patient with a healthy individual of similar age and gender. Each participant provided a 5 ml blood sample, from which plasma was separated and stored at -80 °C for future analysis. The blood samples were collected in K3EDTA tubes, which effectively blocks the coagulation cascade. The study protocol received approval from the Ethics Committee of Golestan University of Medical Sciences (IR.GOUMS.REC.1401.562), and all participating patients provided informed consent. All methods were performed in accordance with the principles outlined in the Declaration of Helsinki (Fig. 1).

### RNA extraction

For the isolation of total RNA from the blood samples, TRIzol (Sinnaclon, Iran) was employed following the manufacturer’s instructions. After blood collection, 1 ml of TriZol were added to 500 ul of each sample was used for RNA isolation. RNA concentration and purity were assessed using a Nanodrop spectrophotometer, with all samples meeting quality thresholds (A260/A280 = 1.8–2.0; A260/A230 = 2.0–2.2; concentration > 100 ng/µL). Furthermore, the quality and integrity of the RNA samples were assessed using a 1% agarose gel electrophoresis. The isolated RNAs were then stored at -80 °C to prevent degradation until they were ready for subsequent use.

### Removal of genomic DNA and cDNA synthesis

The DNA contamination was removed from the extracted total RNA using DNase reagent (Sinnaclon, Iran). After the DNA removal step, cDNA synthesis was conducted using 1 µl of total RNA and a cDNA synthesis kit. The SinaClon First Strand cDNA Synthesis Kit (Sinnaclon, Iran) was utilized for reverse transcription, following the manufacturer’s instructions. The process involved the use of a stem-loop specific primer^37^, for the synthesis of cDNA from the RNA template.

### Quantitative real-time PCR (RT-qPCR)

In the real-time PCR reaction, 1 µl of the synthesized cDNA was used as the template. The PCR reaction employed the RealQ Plus 2x Master Mix Green (Ampliqon, Denmark) as the PCR reagent. For normalization purposes, the internal control gene U6 was utilized. U6 is a commonly used reference gene in gene expression studies, serving as a stable control for normalization of the target gene expression levels. The primers for amplifying the target genes were designed using various tools, including Oligo 7 software, Protocol Online, and the OligoAnalyzer Tool. To ensure primer specificity, a BLAST analysis was performed (Table 1). The qRT-PCR was conducted using the LineGene K system (Bioer, China). In each reaction, the total volume was 10 µl. The components included 5 µl of RealQ Plus 2x Master Mix Green, 0.3 µM of each primer, and 1 µl of cDNA. The cycling conditions were as follows: an initial denaturation step at 95 °C for 15 min, followed by 40 cycles of denaturation at 95 °C for 15 s, and annealing at 61 °C for 20 s.

**Table 1:** Primer sequences utilized in Real-Time quantitative PCR for gene expression Analysis.

	Forward (5’-3’)	Reverse (5’-3’)
5’tRF-His-GTG	ATCTGCCGTGATCGTATAGTGGTT	ACGTGTGCTCTTCCGATCTCG
U6	GCTTCGGCAGCACATATACTAAAAT	CGCTTCACGAATTTGCGTGTCAT

### Statistical analysis

The CT values obtained from RT-qPCR were utilized in the 2^(-ΔΔCT) method to calculate the relative fold expression change. GraphPad PRISM version 8 was used for the statistical analysis. The relative expression of tRF-32-XSXMSL73VL4YK was compared between the patients and controls using an independent t-test. To assess the normal distribution of samples, we applied the Shapiro-Wilk test. Consequently, the independent t-test was utilized to examine the relationship between gene expression levels and clinicopathological characteristics. The expression of candidate tRF in this study had a normal distribution. p-value less than 0.05 was considered statistically significant.

## Results

### In silico predictions showed the structure, genomic locations, and modifications

**Fig. 2:**
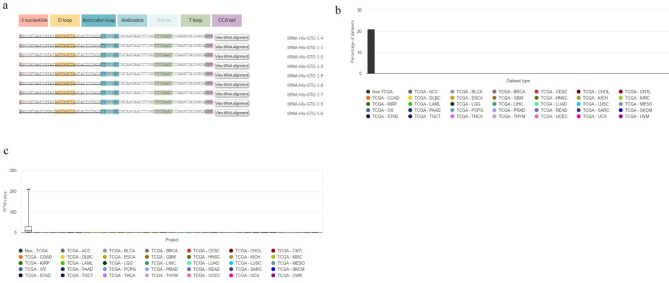
**(a)** mature tRNA sources of the candidate 5’tRF-His-GTG **(b)** Datasets with RPM ≥ 1.0) from TCGA of the candidate 5’tRF-His-GTG **(c)** Range of RPM values per dataset type (From MINTBase V2.0).

After screening all characterized tRFs derived from tRNA-His, a specific tRF, namely tRF-32-XSXMSL73VL4YK, was selected for further analysis. The selection was based on its minimal modifications, which would likely minimize interference during the assessment of its expression levels using qRT-PCR. Furthermore, literature review analysis revealed that tRFs originating from tRNA-His such as tRF-32-XSXMSL73VL4YK have been implicated in crucial functions in cancer physiopathology. Moreover, we performed a comprehensive analysis of tRF-32-XSXMSL73VL4YK using MINTbase v2.0. We focused on determining the genomic loci associated with this particular tRF. The results of our analysis include the number and specific locations of all genomic loci (Table 2). We found that tRF-32-XSXMSL73VL4YK is present at nine genomic locations and occupies an exclusive tRNA space (Fig. 2). To gain further insights into the relationship between tRF-32-XSXMSL73VL4YK and cancer, we explored the TCGA module within MINTbase. The TCGA module provides cancer-specific information by analyzing data from 32 different TCGA cancer types. However, it is essential to note that tRF-32-XSXMSL73VL4YK currently lacks any reported databases within the TCGA dataset, but the expression of this particular tRF has been reported in other databases apart from TCGA (Fig. 2).

It is necessary to remove specific modifications, specifically m1A, m1G, and m3C, as they have the potential to interfere with the process of reverse transcription^38^. Therefore, it was necessary for us to determine if the candidate 5’tRF-His-GTG had any RNA methylation modifications. Our analysis, which involved consulting the MODOMICS database^39^ and reviewing experimental evidence^33^ revealed that the tRF-32-XSXMSL73VL4YK did not possess any methylation modifications (Table 3). Therefore, there were no chemical modifications that could interfere with reverse transcription reaction and consequently PCR amplification.

Furthermore, to gather this information, we conducted searches in the RNA structure section of the MODOMICS database (with 1925 different RNA sequences and 433 different RNA modified residues) using specific queries, including RNA type “tRNA,” Subtype “His,” Ensemble “Eukaryota,” and Organism “Homo sapiens.” This allowed us to retrieve relevant data on the types and positions of modifications associated with the 5’tRF-His-GTG. The specific details concerning the types and positions of modifications on 5’tRF-His-GTG can be found under the ID number 449 and SOTerm SO:0000262 within the database. This finding suggests that demethylating tRF-32-XSXMSL73VL4YK before performing reverse transcription is unnecessary because the fragment does not originate from the regions of tRNA-His that are subject to methylation modifications (Table 3). Furthermore, it is worth noting that while the upregulation of tRF-32-XSXMSL73VL4YK has reported in BBcancer, there are no reports available in the BBcancer database regarding the expression of tRF-32-XSXMSL73VL4YK in blood samples from breast cancer patients.

**Table 2:** The information of 5’tRF-His-GTG in MINTbase.

MINTbase ID	Genomic Locations	Fragment Length	MINTbase Alternative IDs	Anticodon	Exclusive to tRNA space?
tRF-32-XSXMSL73VL4YK	9	32	trna111_HisGTG_1_-_147774845_147774916@-1T.31.32 trna118_HisGTG_1_-_145396881_145396952@-1T.31.32 trna16_HisGTG_1_+_146544773_146544844@-1T.31.32 trna21_HisGTG_1_+_147753471_147753542@-1T.31.32 trna1_HisGTG_15_+_45493349_45493420@-1T.31.32 trna8_HisGTG_15_-_45492611_45492682@-1T.31.32 trna9_HisGTG_15_-_45490804_45490875@-1T.31.32 trna33_HisGTG_6_+_27125906_27125977@-1T.31.32 trna7_HisGTG_9_-_14433938_14434009@-1T.31.32	His-GTG (n)	Yes

**Table 3:** The known position of modifications on tRNA-His based on MODOMICS database

Position in the alignment	0	13	16	20	20 A	32	45B	49	55	58
**Position in sequence**	1	14	17	20	21	33	48	49	55	58
**Modification positions**	xG	Ψ	D	D	D	Ψ	m5C	m5C	Ψ	m1A

**Abbreviations**: xG: unknown modified guanosine Ψ: pseudouridine D: dihydrouridine m5C: 5-methylcytidine m1A: N 1-methyladenosine.

**Table 4:** The relationship between tRF-32-XSXMSL73VL4YK and the clinicopathological characteristics.

Characteristics	*n*	Mean ± SD	*P* value *
Age			
≥ 50	16 (57.1%)	2.10 ± 0.62	0.3162
< 50	12 (42.9%)	1.90 ± 0.39	
**Tumor Size (cm)**			
≥ 4	5 (17.8%)	2.03 ± 0.57	0.7469
< 4	23 (82.2%)	1.95 ± 0.39	
**TNM stage**			
Ⅰ-Ⅱ	17 (73.9%)	2.08 ± 0.66	0.7705
Ⅲ-Ⅳ	6 (26.1%)	2.02 ± 0.25	
**HER2**			
Positive	12 (42.9%)	1.87 ± 0.33	0.2147
Negative	16 (57.1%)	2.12 ± 0.64	
**ER**			
Positive	24 (85.7%)	2.01 ± 0.58	0.9154
Negative	4 (14.3%)	2.03 ± 0.28	
**PR**			
Positive	22 (78.5%)	1.99 ± 0.60	0.4623
Negative	6 (21.4%)	2.12 ± 0.26	
**P53**			
Positive	4 (28.6%)	2.11 ± 0.30	0.6042
Negative	10 (71.4%)	2.01 ± 0.30	
**Ki67**			
≥ 15%	21 (75%)	2.04 ± 0.59	0.5918
< 15%	7 (25%)	1.93 ± 0.35	

* The independent t-test was employed to assess the relationships between gene expression levels and clinicopathological characteristics.

### Upregulation of tRF-32-XSXMSL73VL4YK in breast cancer patients compared to normal controls

**Fig. 3:**
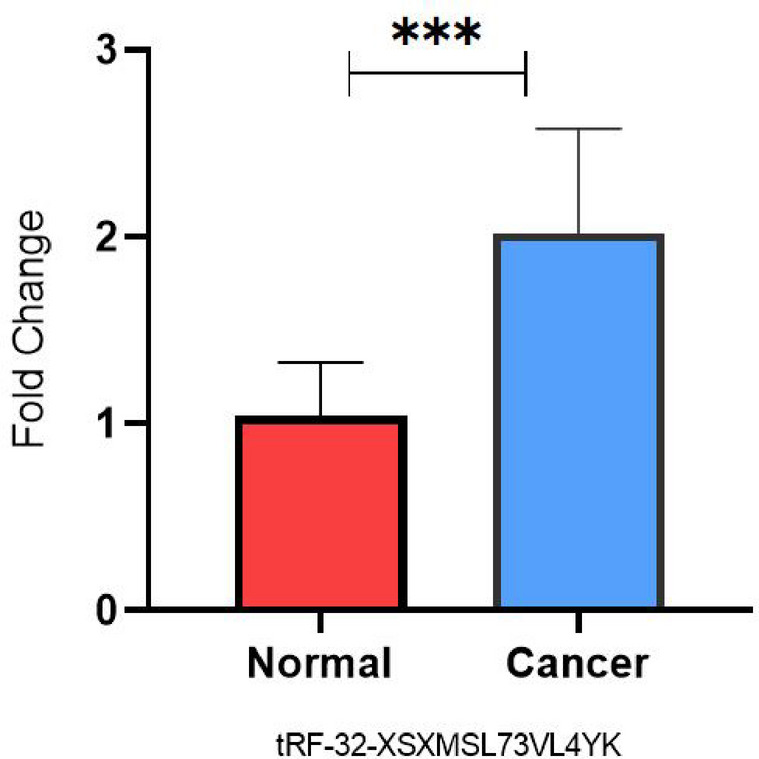
The increased expression of tRF-32-XSXMSL73VL4YK in the patients (*** *p* < 0.001).

We conducted qRT-PCR to quantify the expression levels of tRF-32-XSXMSL73VL4YK in each sample. Our analysis unveiled a considerable upregulation of tRF-32-XSXMSL73VL4YK in breast cancer patients when compared to the normal control group (*p* < 0.001). The expression level of tRF-32-XSXMSL73VL4YK in breast cancer samples was found to be approximately two times higher compared to that in the normal control samples (Fig. 3).

### The relationship between tRF-32-XSXMSL73VL4YK and clinicopathological characteristics

We conducted further investigations to explore the relationship between the expression levels of tRF-32-XSXMSL73VL4YK and various clinicopathological factors in breast cancer patients. Our results revealed that there was no significant association between the expression of tRF-32-XSXMSL73VL4YK and factors such as age (*P* = 0.3162), tumor size (*P* = 0.7469), TNM stage (*P* = 0.7705), as well as commonly used immunohistochemical markers for breast cancer prognosis and therapy, including ER, HER2, Ki-67, PR, and p53 (Table 4).

## Discussion

Breast cancer is a heterogeneous disease that can result from various distinct genetic and epigenetic alterations in mammary epithelial cells^40^. Early diagnosis is crucial for effective treatment and favorable prognosis, as patients with smaller tumors have significantly higher survival rates and lower mortality^9^.Advances in high-throughput sequencing technologies have led to the identification of new classes of small non-coding RNAs, including functional tRFs, in various cancers such as breast, gastric, and colorectal cancers ^41^.

One such fragment, tRF-32-XSXMSL73VL4YK, is a 5’-tRF derived from tRNA-His and is primarily located in the cytoplasm. In this study, we observed a significant upregulation of tRF-32-XSXMSL73VL4YK in blood samples from breast cancer patients, suggesting its potential as a biomarker to distinguish between patients and healthy individuals. We further examined the relationship between tRF-32-XSXMSL73VL4YK expression and clinicopathological characteristics across 28 samples, but found no significant correlation. While TCGA offers extensive cancer-specific transcriptomic data, we found that tRF-32-XSXMSL73VL4YK was not annotated or reported within TCGA datasets. This absence is likely due to TCGA’s primary focus on coding and well-characterized non-coding RNAs, whereas many recently identified tRFs, including tRF-32-XSXMSL73VL4YK, have yet to be systematically integrated into TCGA pipelines. However, the expression and biological relevance of tRF-32-XSXMSL73VL4YK have been supported by alternative studies, such as GSE123967 ^34^, which validate its presence and potential role in cancer-related pathways.

Our findings align with those of Wang et al., who also reported significant upregulation of tRF-32-XSXMSL73VL4YK in breast cancer tissues compared to adjacent healthy tissues (34). The enrichment analysis of tRF-32-XSXMSL73VLAYK revealed its involvement in key biological processes, including neurogenesis, neuron differentiation, and neurological function (GO:0008070, GO:0045666, GO:0001664), as well as transcriptional and vascular regulation (GO:0004244, GO:0005116). Cellular component analysis links it to the Golgi apparatus, ER-Golgi trafficking (GO:0005794, GO:0033116), and neuronal structures (GO:0097488), while molecular functions highlighted roles in transcriptional regulation (GO:0001228, GO:0000982), microtubule binding (GO:0008017), and cytoskeletal interactions (GO:0008092). KEGG pathway associations further connect it to neurotransmission (e.g., dopaminergic synapses) and metabolic pathways ^34^. Together, these findings suggest tRF-32-XSXMSL73VLAYK regulates neuronal development, cellular trafficking, and metabolic homeostasis, with potential implications in neurological and degenerative diseases.

Similarly, increased expression of another tRF, 5’tiRNA-His-GTG, has been reported in colon cancer tissues. In vitro and in vivo studies support its oncogenic role in colon cancer. Mechanistically, its biogenesis is influenced by hypoxic conditions in the tumor microenvironment and is regulated by the HIF1α/angiogenin (ANG) axis. 5’tiRNA-His-GTG targets LATS2, thereby inhibiting the Hippo signaling pathway and promoting the expression of genes involved in cell proliferation and anti-apoptotic processes ^33^. Moreover, Tang et al. reported a significant decrease in the expression of a specific 5’-tRF-His in breast cancer tissues and serum, which was associated with increased lymph node metastasis. This tRF was found to regulate breast cancer cell proliferation and apoptosis via the Pan-AGO protein and to inhibit tumor growth in vivo. The study also identified the 5’-tRF-His/CKAP2/Erk2 axis as a key regulator of these processes, suggesting its potential as a serum-based tumor marker ^32^. Additionally, tRF3E is a circulating tRF which found in the bloodstream of HER2-positive breast cancer patients. The highest levels of tRF3E were observed in healthy controls, with a marked decline in early-stage patients and the lowest levels detected during metastasis. These findings suggest a significant inverse correlation between plasma tRF3E levels and tumor progression in HER2-positive breast cancer, reinforcing its potential as a diagnostic and prognostic biomarker ^42^. 

## Limitations

Our study has several limitations that should be acknowledged. First, the sample size was relatively small, which may limit the statistical power and generalizability of our findings. Given the small sample size, performing a ROC curve analysis could potentially lead to overestimation of diagnostic accuracy, resulting in falsely high sensitivity and specificity. Expanding the cohort in future studies would strengthen the reliability of the results and help uncover potential associations not evident in a smaller sample. Notably, the absence of a significant relationship between the dysregulation of tRF-32-XSXMSL73VL4YK and clinicopathological characteristics suggests the need for larger populations to better explore possible links between this tRF and specific tumor features. 

Moreover, comparative analyses with established diagnostic markers are warranted to evaluate the clinical performance and utility of tRFs. Such comparisons would help clarify the advantages, limitations, and potential synergistic roles of tRFs in diagnostic and prognostic applications. To further enhance the robustness of our findings, the integration of bioinformatics and machine learning approaches is recommended. These computational tools can assist in identifying tRF-based biomarker signatures and improve the accuracy of breast cancer diagnosis and prognosis predictions.

It is also important to note that functional validation and mechanistic studies are necessary to elucidate the biological significance of tRFs in breast cancer development and progression. Understanding the underlying molecular pathways and interactions will provide a stronger foundation for considering tRFs as clinically relevant biomarkers. In particular, investigating the functional role of tRF-32-XSXMSL73VL4YK and its molecular targets may yield insights into its potential applications in diagnosis, prognosis, or therapy. Addressing these limitations through larger cohorts, comparative marker analysis, advanced computational modeling, and in-depth mechanistic studies will be essential to fully validate and establish the role of tRF-32-XSXMSL73VL4YK in breast cancer research and clinical practice.

## Conclusion

In conclusion, we identified a 5′tRF-His-GTG, designated tRF-32-XSXMSL73VL4YK, as a significantly upregulated fragment in the blood of breast cancer patients, indicating its potential relevance in disease biology. However, no significant associations were observed between the dysregulation of tRF-32-XSXMSL73VL4YK and clinicopathological features such as age, tumor size, TNM stage, or IHC markers. This lack of association with clinicopathological characteristics suggests that tRF-32-XSXMSL73VL4YK may not serve as a standalone diagnostic marker for breast cancer but holds promise as a non-invasive prognostic biomarker or as part of a multi-marker panel to improve early detection and patient stratification. Future mechanistic studies are necessary to elucidate the role of this tRF in tumor biology and assess its potential clinical applications. Future studies should consider analyzing breast tissue samples, which may provide more direct insights into tumor-specific expression patterns compared to blood-based biomarkers. Evaluating matched tumor and adjacent normal tissues could help clarify its functional relevance in breast cancer. Additionally, integrating multi-omics approaches may uncover underlying mechanisms and enhance our understanding of its diagnostic and prognostic utility.

## Data Availability

All data generated or analyzed during this study are included in this Manuscript.
